# Sub-Nanowatt Ultrasonic Bio-Telemetry Using B-Scan Imaging

**DOI:** 10.1109/OJEMB.2021.3053174

**Published:** 2021-01-20

**Authors:** Sri Harsha Kondapalli, Shantanu Chakrabartty

**Affiliations:** Department of Electrical and Systems Engineering at Washington University in St. Louis, St. Louis, MO 63130 USA

**Keywords:** B-Scan imaging, echoscope, low-power communication, ultrasound telemetry

## Abstract

**Goal:**

The objective of this paper is to investigate if the use of a B-scan ultrasound imaging system can reduce the energy requirements, and hence the power-dissipation requirements to support wireless bio-telemetry at an implantable device.

**Methods:**

B-scan imaging data were acquired using a commercial 256-element linear ultrasound transducer array which was driven by a commercial echoscope. As a transmission medium, we used a water-bath and the operation of the implantable device was emulated using a commercial-off-the-shelf micro-controller board. The telemetry parameters (e.g. transmission rate and transmission power) were wirelessly controlled using a two-way radio-frequency transceiver. B-scan imaging data were post-processed using a maximum-threshold decoder and the quality of the ultrasonic telemetry link was quantified in terms of its bit-error-rate (BER).

**Results:**

Measured results show that a reliable B-scan communication link with an implantable device can be achieved at transmission power levels of 100 pW and for implantation depths greater than 10 cm.

**Conclusions:**

In this paper we demonstrated that a combination of B-scan imaging and a simple decoding algorithm can significantly reduce the energy-budget requirements for reliable ultrasonic telemetry.

## I. Introduction

Existing clinical and FDA approved bench-top ultrasound systems are now able to generate real-time, high-resolution images at rates greater than 10 000 frames per second [1]. These commercial systems are also flexible enough that users can adjust different imaging parameters like the imaging depth, the aperture size, and the receiver sensitivity under program control. Also, these platforms can support computationally intensive image processing algorithms that have been used to enhance the ultrasound image frames in real-time [2]. The large data acquisition and computational bandwidth that is afforded on these ultrasound imaging systems could potentially be used for designing energy-efficient in-vivo telemetry links in addition to using the system for conventional diagnostic imaging. The use of FDA-compliant ultrasound readers will also simplify the future adoption of the telemetry technology by practicing clinicians and researchers, without the need to develop a custom wireless telemetry system/device.

In literature, in-vivo telemetry links have been implemented using radio-frequency (RF) and ultrasound modalities [3]–[6]. However, RF-based telemetry links can only be supported at limited implantation depths due to constraints on the antenna size (in the case of high-frequency RF) or due to attenuation losses (in the case of ultra-high frequency RF). Ultrasound telemetry links, on the other hand, can be supported for implantation depths greater than 10 cm, and thus have been proposed for many biomedical applications such as neural recording and stimulators [7], cardiac pacemakers [8] and monitoring bone healing [9]–[11].

In our previous work [12], we demonstrated that a commercial-of-the-shelf (COTS) M-scan ultrasound imaging system could be used for designing multi-access in-vivo ultrasound communications links. We demonstrated data rates up to 800 Kbps for implantation depths greater than 12 cm while dissipating only microwatts during transmission. However, if the transmission energy-budget at the implant could be reduced down to nano-watts, not only the battery form factor at the implant could be reduced but the telemetry could potentially be self-powered using energy harvested in-vivo [13]. Reducing the transmission power will also reduce imaging artifacts when the ultrasound system is used simultaneously for imaging other physiological processes like blood flow or tissue palpitations [14]. In this paper, we investigate if the transmission energy requirements at the implant can be reduced by exploiting the beam-forming feature available on most commercial B-scan ultrasound imaging systems.

**Fig. 1.**
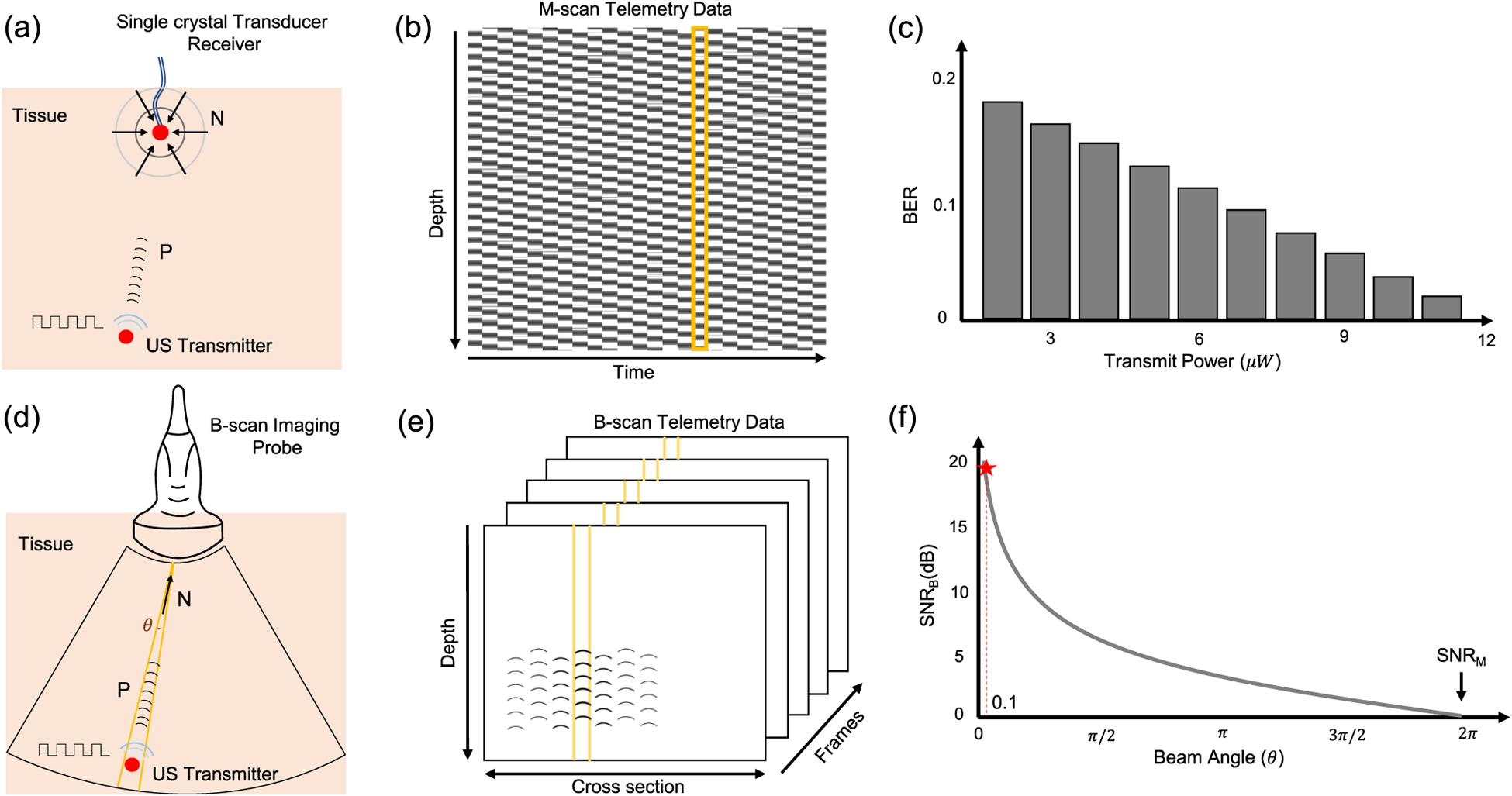
(a) Principle of M-scan telemetry as reported in [12]; (b) Illustration of a typical M-scan image when the data is being transmitted using ON-OFF signaling. (c) BER corresponding to the M-scan telemetry link as reported in [12] when the transmit power is varied from 1 *μ* W to 12 *μ*W. (d) Principle of the proposed B-scan telemetry. (e) Illustration of a typical B-scan image showing the presence of transmitted data in a 2D cross-section as well as across several B-scan frames. (f) Anticipated improvement in SNR (highlighted by star) for B-scan telemetry compared to M-scan telemetry scheme when operated around beam-angle (*θ*) of 0.1 radians (≈ 5°). Note that P and N are the corresponding signal and noise power in the received signal captured by the imaging probe.

Fig. 1 shows the principle underlying the proposed B-scan approach and compares it against our previously reported M-scan based ultrasound telemetry. In M-scan telemetry shown in Fig. 1(a), a communication link is established between two single-element ultrasound crystals. One of the elements acts as a transmitter and is implanted inside the tissue. The other element, acting as an interrogator is located on the surface of the skin and is driven by an M-scan ultrasound imaging system, as shown in Fig. 1(a). Fig. 1(b) shows a sample M-scan image data when the transmitter crystal is driven by a periodic ON-OFF signal. The transmitted pulses create an imprint on the M-scan image, which upon further processing reveals the transmitted data. In [12], we have characterized the M-scan telemetry link in terms of its BER and the transmission power. Fig. 1(c) shows a sample result which shows that only microwatts of transmission power are required to achieve reasonable BER. However, due to lack of directionality the received noise power (N) is isotropic as shown in Fig. 1(a). In practice, the information about the location of the implant is known a-priori and hence, could be leveraged to boost the received signal to noise power ratio (SNR) using beam-forming. In the proposed B-scan telemetry, a linear array imaging probe (interrogator) is used for beam-forming and for capturing the transmitted data from the directions of interest and a specific depth, as shown in Fig. 1(d). Sample B-scan data is shown in Fig. 1(e) where the effective SNR (*SNR*_*B*_) is determined by the ratio of received signal power (P) and the noise incident (N) from a specific beam-angle (*θ*). Similar to SNR calculations used in radio-frequency multiple-input multiple-output (MIMO) systems [15], it is anticipated that the effective SNR (*SNR*_*B*_) for B-scan telemetry should be inversely proportional to the interrogation beam angle. Thus, the proposed B-scan based telemetry should provide higher SNR compared to the M-scan telemetry.

The main contributions of this paper are as follows:

- We extend the framework of imaging-based telemetry for the case of B-scan imaging system that can be used for designing reliable ultrasonic communication links with a millimeter-scale implanted transducer.
- We present a computationally light method to decode the information embedded in the B-scan images and algorithms to characterize the communication link in terms of its BER and transmit power.
- We investigate the limits on transmission power that can support reliable ultrasonic telemetry.

## II. Materials and Methods

**Fig. 2.**
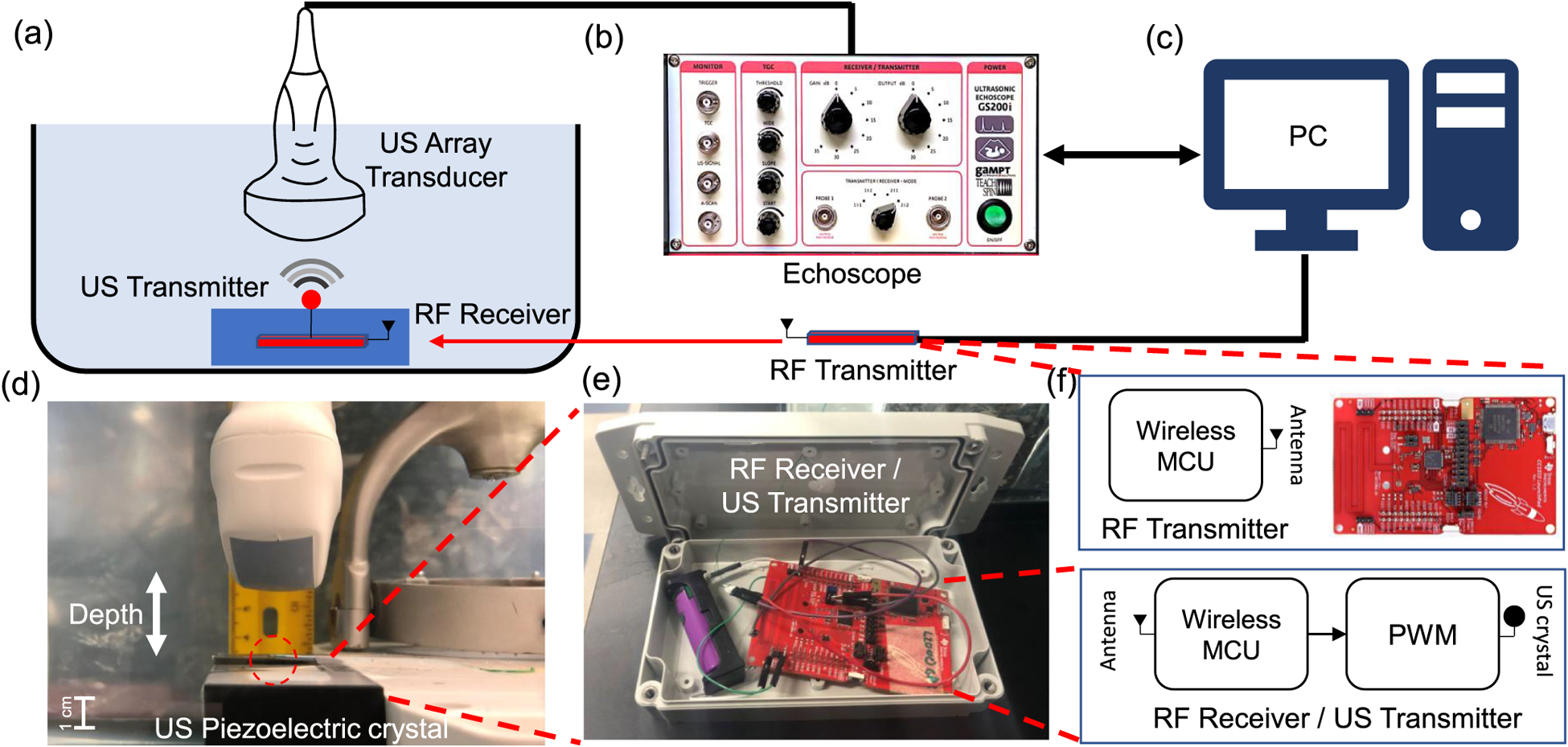
(a) Illustration of the experimental setup used to verify and quantify the performance of B-scan telemetry in a water transmission medium. A linear transducer array is driven by (b) an echoscope which is used to acquire B-scan images which are then post-processed on a (c) computer; (d) Picture of the water-bath highlighting the interrogation depth and the relative orientation of the imager and the implant; (e) The prototype of the ultrasound transmitter implemented using a TI CC1310 board and enclosed in a sealed waterproof container. (f) RF module is used for remote programming of the submerged ultrasound.

Fig. 2 shows the experimental setup that has been designed to verify and characterize the proposed B-scan telemetry. An ultrasound transmitter was prototyped using a Texas Instruments microcontroller board (TI CC1310) and the system was packaged in a water-proof container and then suspended in a water bath as shown in Fig. 2(a). A millimeter-sized piezoelectric crystal was used as an ultrasound transducer(Sonometrics Corp.) and was fixated outside the container such that it was aligned with the ultrasound imaging probe, as shown in Fig. 2(a). The B-scan data acquired by a commercial echoscope was stored on a computer (Fig. 2(c)) and the data is post-processed using methods described in Section II-E. An external RF micro-controller platform (TI CC1310) was used to wirelessly control the power and transmission rate of the ultrasound transmitter submerged in the water-bath, as shown in Fig. 2(f). Note that the RF link was only turned on for reprogramming the ultrasound transmitter to transmit at different power levels or for adjusting the transmission rate. At all other times, the RF link was disabled to minimize electromagnetic coupling.

### A. Characterization of the Ultrasound Transmitter Crystal

**Fig. 3.**
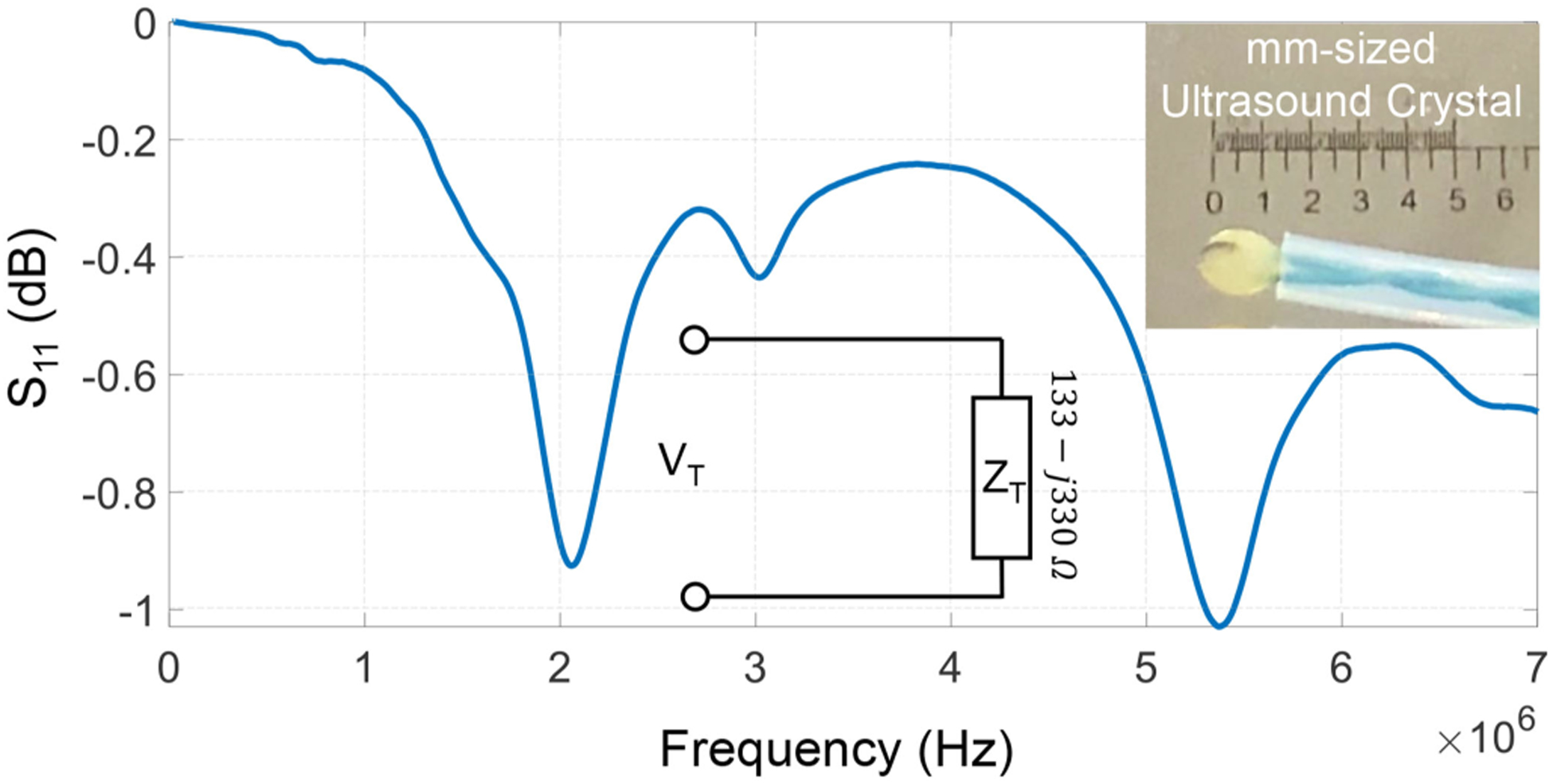
Measured *S*_11_ characteristics of the ultrasound transmitter crystal shown in the inset.

Fig. 3 shows the measured *S*_11_ characteristic of the piezoelectric crystal used as an ultrasound transmitter. The result shows that the crystal can be driven at different resonances but at the operating frequency of 2 MHz, the crystal impedance was measured to be 133 − *j*330 Ω. The estimated impedance has been used in the later sections to calculate the transmitted power using an approach similar to the method described in [12]. The crystal is driven by an oscillator module integrated on the microcontroller board and the amplitude of the driving signal can be adjusted from *V*_*T*_ = 0–5 V, in steps of 1.2 mV. The voltage sweep translates to sweeping the transmit power from 0 – 0.7*μW* at sub-*nW* resolution. Note that the attenuation coefficient of ultrasound in water is given by 0.0022 dB/MHz/cm which leads to a 90 – 97% efficiency in receiving the signal-power at the imager/receiver when the distance to the transmitter is varied from 10 cm to 2 cm.

### B. Echoscope and Linear Transducer Array

The B-scan ultrasound imaging system was implemented by interfacing a convex array ultrasound probe (C5-2R60S-3) to a commercial echoscope (gaMPT GS200i with an integrated Telemed MicrUs ultrasound system). The 256-element ultrasound probe used in our experiments has a field view of 60 degrees with a 65 mm radius of curvature and its operating frequency can be adjusted from 2 MHz to 5 MHz. This focus of the system is customizable with a variable frame-rate of 13–24 fps while being able to image at depths of 90–230 mm respectively. Data from the echoscope was acquired via a USB 2.0 interface and the post-processing of the data was performed in MATLAB©. For the experiments, the echoscope was set to a low-transmit power setting and high-receive gain setting to reduce diagnostic imaging artifacts.

### C. RF Triggering, Control and Programming

The submerged ultrasound transmitter uses an integrated 915 MHz RF module to communicate with another RF transmitter located outside the water-bath. The RF link was used to wirelessly adjust different transmit parameters (transmission rate and transmission power) without physically disturbing the submerged transmitter. To overcome RF attenuation losses in the water-medium, we set the RF to transmit power to the maximum allowed level while operating within the 915 MHz ISM frequency band. Both the RF transmitter and the submerged RF receiver were designed using the TI CC1310 wireless microcontroller platform. In addition to the TI radio-module, the submerged RF receiver also had a pulse width modulator (PWM) that was used for driving the ultrasound transmitter crystal. The RF transmitter located outside the water-bath was controlled using a serial link to a computer which was also used to send information about the pulse width, pulse rate, and transmit power. A transmitter was packaged inside a sealed container which was also used to stabilize the piezoelectric crystal at the bottom of the water tank, as shown in Fig. 2(d).

### D. Data Collection and Processing

**Fig. 4.**
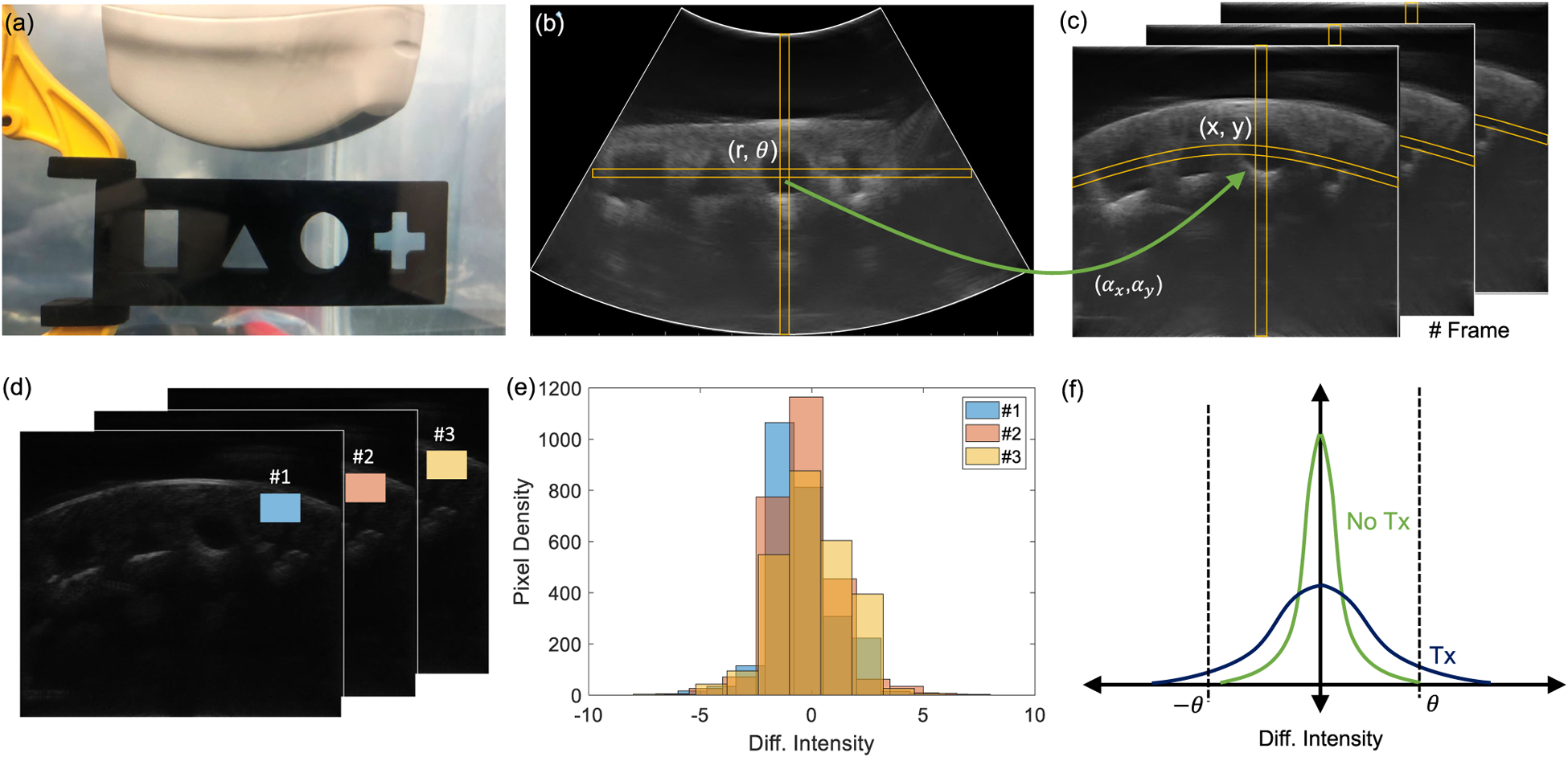
Illustration of the B-scan image transformation steps using a (a) template imaging stub where the original B-scan image (b) is transformed pixel-wise into Cartesian coordinates (c); (d) Difference image recovered by high-pass filtering the B-scan image frames; (e) Pixel intensity distributions estimated for a given frame at different instances of time. (f) Anticipated change in pixel intensity distributions in the presence and absence of transmission signal.

In this sub-section, we illustrate the data collection and processing steps using B-scan images of a stub comprising of engravings of different geometric shapes, as shown in Fig. 4(a). As shown in Fig. 4(b), the axial direction to the ultrasound imager provides information about the variations in acoustic impedance along with the depth and the lateral direction shows the variations in the acoustic impedance of the medium parallel to the imaging plane. Every frame consists of multiple vertical lines which correspond to a pulse-echo interrogation cycle acquired by the linear array transducer. Echowave II software and user interface provided with the echoscope were used to adjust the transmit and the receive gain of the echoscope and the acquired B-scan images were saved in a PNG format. Note that the acquired images are represented in polar coordinates and the area under interrogation is highlighted using white borders in Fig. 4(b). To translate the image pixels into a Cartesian coordinate system, each PNG image was converted to grayscale after which a linear mapping is applied, as illustrated in Fig. 4(b)–(c). If a pixel location in the polar coordinates is represented by (*r*, *θ*), then its corresponding location in the Cartesian coordinate is given by the mapping

(1) $$x=\left\lfloor r\;\text{sin}\theta +\alpha_{x}\right\rfloor$$

(2) $$y=\left\lfloor r\;\text{cos}\theta +\alpha_{y}\right\rfloor .$$

Note that ⌊.⌋ are floor operators, and (*α*_*x*_, *α*_*y*_) is an offset as shown in Fig. 4(b). This transformation also reduces the image size to (*x*_*max*_, *y*_*max*_) = 530×600 pixels as shown in Fig. 4(c). In the transformed image, if $U_{\left(x,y\right)}^{i}$ denotes the pixel intensity at location *x*, *y* on the *i*^*th*^ frame, a difference operation is applied according to

(3) $$W_{\left(x,y\right)}^{i}=U_{\left(x,y\right)}^{i}-U_{\left(x,y\right)}^{i-1}.$$

This operation eliminates the static background and retains only information that fluctuates at frequencies greater the frame-rate. Fig. 4(d) shows the filtered data *W* which has been appropriately scaled to improve visualization. Distribution of pixel intensities within an image patch is then estimated across different frames, as shown in Fig. 4(e). The result for each frame follows an approximate bell-shaped curve, as shown in Fig. 4(f), indicating the distribution can be assumed to be quasi-stationary to the image frames. When the transmitter is ON, we expect the distribution of *W* to change such that the statistical variance of *W* should increase, as shown in Fig. 4(f). The challenge in this case would be to design a simple and yet robust decoding algorithm that can detect this change or increase in variance.

### E. Telemetry Decoding Algorithm

When the ultrasound transmitter is ON, the imager captures the changes in the received signal power as changes in the pixel intensities on the B-scan images. This is illustrated for high transmit power levels by measuring the column-wise pixel intensities according to

(4) $$I_{y}^{i}=\frac{1}{x_{max}}\sum_{x}\left|W_{\left(x,y\right)}^{i}\right|$$

where $\vec{I}$ is a vector containing the mean intensity value estimated along the column in the *i*^*th*^ frame.

The statistical properties of $\left|W_{\left(x,y\right)}^{i}\right|$ estimated over in a patch of size *P* × *Q* can be quantified by its mean and standard deviation measures according to

(5) $$\mu_{\left(P,Q\right)}^{i}=\frac{1}{PQ}\sum_{x}^{P}\sum_{y}^{Q}\left|W_{\left(x,y\right)}^{i}\right|$$

(6) $$\sigma_{\left(P,Q\right)}^{i}=\sqrt{\left[\frac{1}{PQ}\sum_{x}^{P}\sum_{y}^{Q}{\left(\left|W_{\left(x,y\right)}^{i}\right|-\mu_{\left(P,Q\right)}^{i}\right)}^{2}\right]}$$

In this work, the algorithm used for decoding the ON (logic level 1) and OFF (logic level 0) states *D*^*i*^ of the transmitter in the *i*^*th*^ frame is given by

(7) $$D^{i}=\{\begin{array}{c} 1\text{ when},\text{ max}\left[W_{{\left(x,y\right)}_{ON}}^{i}\right]>\text{max}\left[W_{(x,y)OFF}^{i}\right]\\ 0\text{ when},\text{ max}\left[W_{{\left(x,y\right)}_{ON}}^{i}\right]\leq \text{max}\left[W_{{\left(x,y\right)}_{OFF}^{i}}\right] \end{array}$$

Based on the decoded bits, the BER corresponding to the B-scan telemetry can be estimated by taking the ratio between the total number of errors and the total number of frames.

## III. Results

**Fig. 5.**
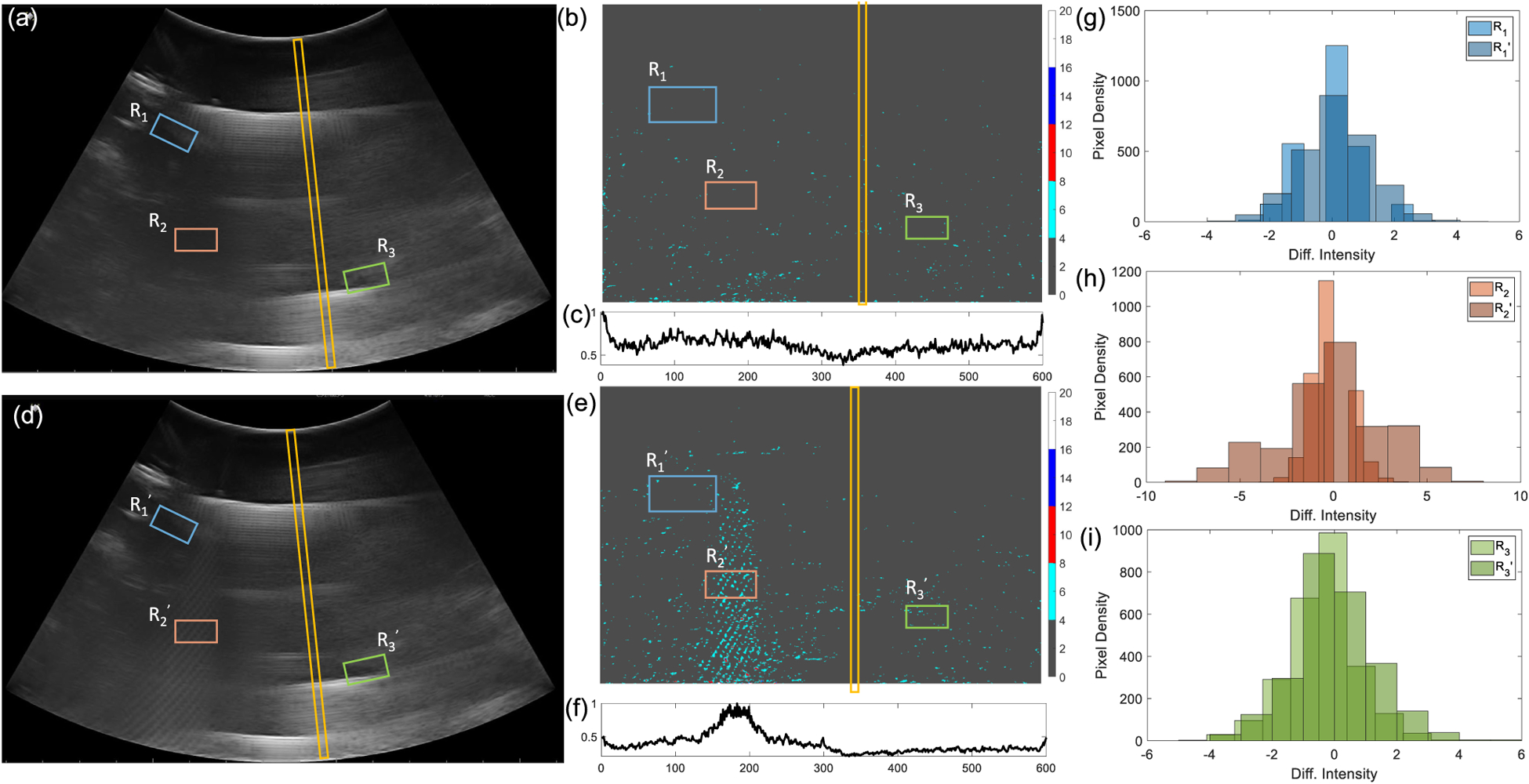
(a) Sample B-scan image frame when the transmitter is OFF and the corresponding transformed image (b) after filtering and the (c) column-wise mean intensity vector *Ī*; (d) Sample B-scan image frame when the transmitter is ON and the corresponding transformed image (e) after filtering and the (f) column-wise mean intensity vector *Ī*; (g)-(i) Pixel intensity distributions corresponding to different image patches (*R*_1_, *R*_2_, *R*_3_) when the transmitter is OFF and ($R_{1}^{\prime }$, $R_{2}^{\prime }$, $R_{3}^{\prime }$) when the transmitter is ON.

The experimental setup shown in Fig. 2 was used to collect B-scan data for two specific cases: (a) when the submerged ultrasound transmitter is OFF (sample scan shown in Fig. 5(a)); and (b) when the transmitter is OFF (sample scan shown in Fig. 5(d)). The respective post-processed scans are mapped into Cartesian coordinates as shown in Fig. 5(b)–(e). Fig. 5(c),(f) shows the corresponding column-wise average intensity ($\vec{I}$ - defined in equation 4), for each of the scans. Fig. 5(g)–(i) compares the distribution of the pixel intensities measured in different patches of the scan (*R*_1_, *R*_2_ and *R*_3_ highlighted in Fig. 5(a) and *R*_1_,′ $R_{2}^{\prime }$ and $R_{3}^{\prime }$ in Fig. 5(e)) for the ON and OFF cases respectively. Note that the distributions for the ON and OFF states measured for the patch *R*_2_ are sufficiently different from each other compared to distributions estimated using the other patches.

### A. Quality Metrics

**Fig. 6.**
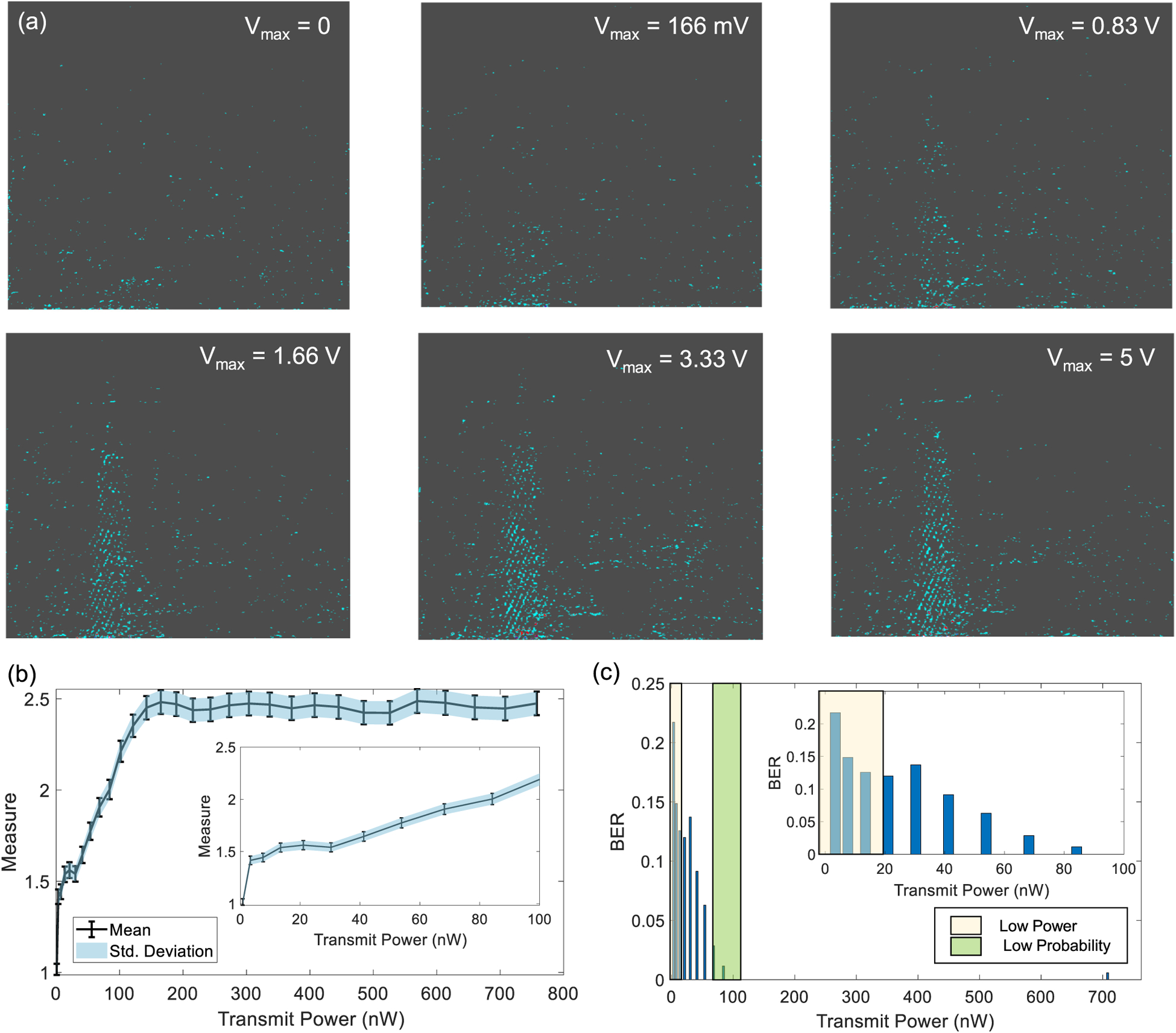
(a) High-pass filtered B-scan image frames measured at different magnitudes of transmitted power; (b) Estimated mean and std. deviation for an image patch for different levels of transmitted power; (c) Estimated BER when the transmitted power is varied from 10 – 700 nW. Inset highlights two operating regions (1–20 nW and 100nW) where sensitivity analysis has been performed.

Several experiments were conducted for different levels of transmit power. Some examples of filtered B-scan data are shown in Fig. 5(a). Once we identified a target patch in the scan we can quantify the quality of the telemetry signal within the patch using the mean absolute intensity and the standard deviation metrics estimated across the pixel intensities. The BER corresponding to the telemetry signal is estimated according to the decoding algorithm in equation 7. The BER metric was then measured for different values of *V*_*max*_ which is the maximum amplitude of the voltage pulse (*V*_*T*_) used to drive the ultrasound crystal at the transmitter. Using the crystal equivalent circuit shown in Fig. 3, the transmitted power can be estimated from *V*_*max*_ as the power dissipated at the equivalent resistor *R*_*T*_ . Fig. 6(b) shows the mean and the standard deviation measured estimated for the image patch for different levels of transmitter power *P*_*T*_. The results show that both the mean and standard deviation of *|W|* increases with the increase in transmitted power. However, note that both the measures saturate at higher transmit power levels which could be due to the saturation of the B-scan amplifier or due to the limited driving capability of the ultrasound crystal.

### B. BER Analysis

Using the decoder defined in equation 7, the transmitted state was estimated which was then used to estimate the BER corresponding to different transmit power levels. The corresponding results are shown in Fig. 6(c). Note that even though the size and the shape of the patch could be varied, we chose a rectangular patch of a specific size that gives the best BER. The BER results in Fig. 6(c) shows that the system performance is optimal when the transmit power level is more than 100 nW and at transmitting power level of 1*nW* the measured BER was as high as 0.25.

### C. Sensitivity Analysis

**Fig. 7.**
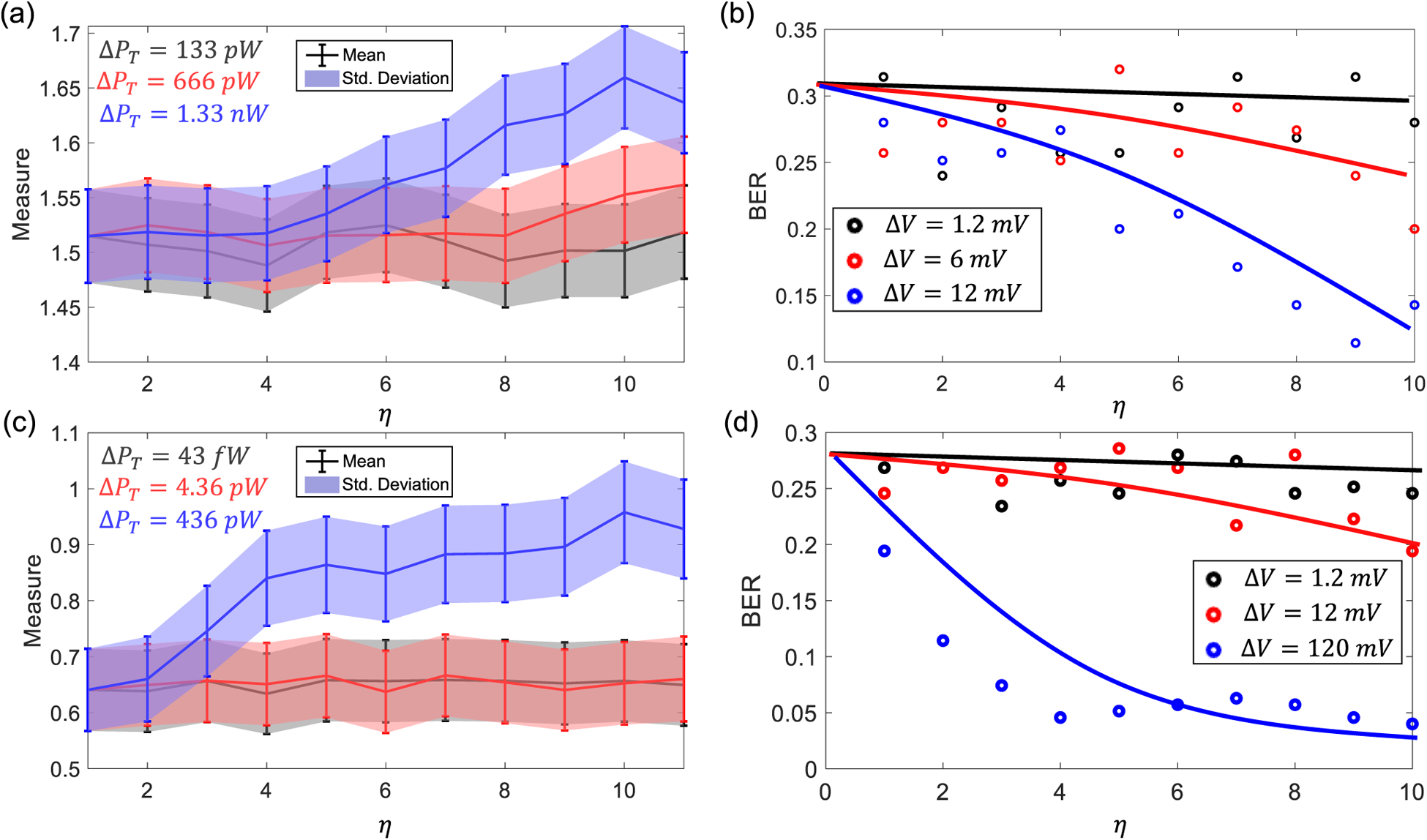
Sensitivity analysis in the low BER region where the transmitted power *P*_*T*_ = 101.5 nW: (a) Mean and std. deviation values of the image patches; (b) the estimated BER when the magnitude of the transmitted pulsed is varied in increments of 1.2 mV-12 mV. Sensitivity analysis in the low BER region where the transmitted power *P*_*T*_ = 0 nW: (c) Mean and std. deviation values of the image patches; (d) the estimated BER when the magnitude of the transmitted pulsed is varied in increments of 1.2 mV-120 mV.

In the next set experiment, we quantified the robustness of the B-scan telemetry system using sensitivity analysis. We characterized the sensitivity for two different regions: (a) when the transmit power *P*_*T*_ was low; and (b) when the system metrics start saturating, as highlighted by the *Low Probability* region in Fig. 6(c). The measured mean and std. deviation are shown in Fig. 7(a) for the case where the magnitude of the driving pulse changed in increments of Δ*V* = [1.2 mV, 6 mV, 12 mV] according to *V*_*max*_ = 1.83 + *η*Δ*V*. The corresponding transmit power variations for the voltage increments of 1.2 mV, 6 mV and 12 mV are Δ*P*_*T*_ = 133 pW Δ*P*_*T*_ = 666 pW and Δ*P*_*T*_ = 1.33 nW respectively. The results show no significant improvement in signal quality measure with increments ranging in Δ*P*_*T*_ = 133 pW but the performance deviation is evident when the transmit power is varied in steps of Δ*P*_*T*_ = 1.4 nW. This effect can also be seen in the BER plot in Fig. 7(b), where the BER change for the case of Δ*V* = 1.2 V is not significant compared to the case Δ*V* = 6 mV and Δ*V* = 12 mV respectively. Similar results corresponding to *V*_*m*_*ax* = 0 are shown in Fig. 7(c). Here the increments in transmitted power Δ*P*_*T*_ = [43 fW, 4.36 pW, 436 pW] correspond to the voltage increments Δ*V* = [1.2 mV, 12 mV, 120 mV]. In this case as well, the mean and std. deviation for this data show no significant improvement in signal quality when the transmit power is increased in steps of Δ*P*_*T*_ = [43 fW, 4.36 pW] but for the later case of Δ*P*_*T*_ = 436 pW significant improvement in signal quality is observed. This effect can also be seen in the corresponding BER plots shown in Fig. 7(d).

### D. Telemetry Driven by Unregulated Power Source

The next set of experiments were designed to verify that B-scan telemetry and the decoding algorithm is useful for applications where an unregulated power source drives the ultrasound transmitter. This scenario arises in self-powered systems [16] where the energy harvested from a transducer is used directly to power the telemetry functions without any voltage regulation. For example in our previous work [13] we showed that cardiac valvular perturbations could be exploited to harvest nano-watts to micro-watts of power using a piezoelectric suture. When an unregulated power source is used, the echoes generated by the ultrasound transmitter are non-harmonic in nature and could be considered as noise.

**Fig. 8.**
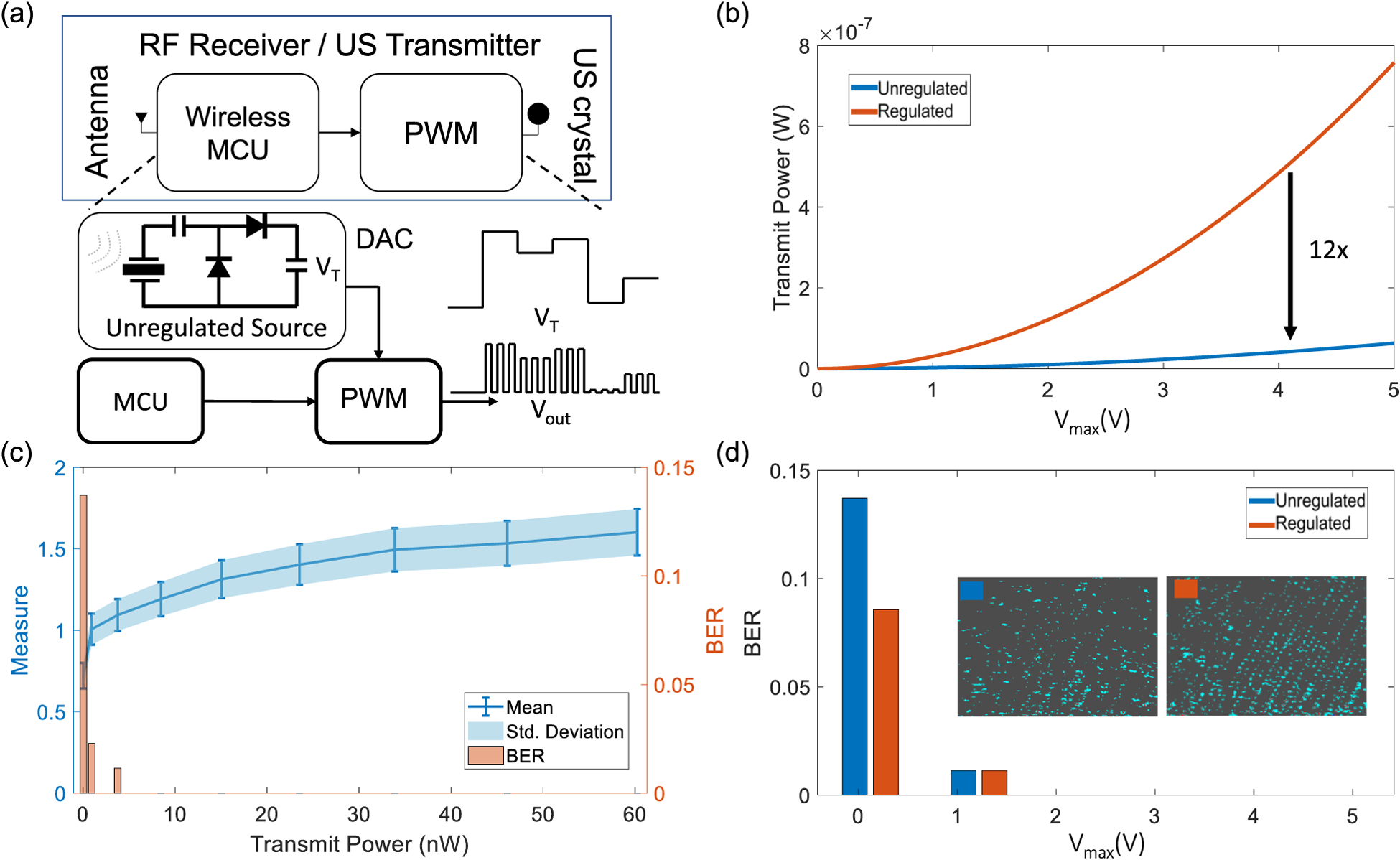
Emulation of the ultrasound transmitter driven by an unregulated power source using (a) programmable DAC and a PWM module; (b) Comparison of estimated transmit power levels for the regulated and the unregulated case; (c) Mean, standard deviation and BER measured for levels of transmit power for the unregulated case; (d) Comparison of the BER for the regulated and the unregulated case for different values of *V*_*max*_.

For this experiment we emulate an unregulated power source using a programmable 12-bit digital-to-analog converter (DAC) that was used to drive the supply of the pulse-width modulator, as shown in Fig. 8(a). Note that the amplitude of the transmitted ultrasound pulses is modulated using a pseudo-random generator that is sampling voltages from the DAC II-C. Also, note that the transmit power for the modulated voltage source needs to be reduced by a factor of 12 (variance of a uniformly distributed noise), when comparing to the case of having a stable voltage source, as shown in Fig. 8 (b). Fig. 8(c) shows the mean and the standard deviation metrics for the noise-modulated ultrasound transmission along with the BER of the decoder estimated at each transmit power level. Note that the decoder BER for this case is similar to the case reported in Fig. 6(c) but at much lower levels of transmit power.

## IV. Discussion

Table I compares this work with the prior work in the area of in-vivo ultrasonic telemetry [12], [17], [18] that highlights the transmit power and normalized power at the implant. Note that the previous works were geared towards improving the data rates and quality of communication link by exploring different modulation schemes. Our reported data rates are limited by the frame rate of the echoscope which is approximately 27 fps. However, these values should scale if a more advanced ultrasound imaging system with higher frame rates is used. Also, note that the field of view and the number of piezoelectric elements available at the linear transducer array will affect the receive beam-angle which in turn will determine the BER of the proposed B-scan telemetry.

Even though our reported telemetry rates and BER are low the transmit power levels are significantly lower. This is important for applications with fixed data-rates or when the implant is located at large depths such as in bone healing applications [24] or spinal-fusion monitoring applications [25]. In these applications, the implant needs to operate at limited energy budgets but the latency in interrogation is not very critical. Also, the use of FDA approved ultrasound imaging systems for telemetry would make the translation of this approach more clinically acceptable.

### A. Performance Gain

Comparing M-scan and B-scan telemetry techniques, we observe two orders of improvement in signal transmit power when results corresponding to low BER (Fig. 1(c) and Fig. 6(c)) is achieved at 12*μW* and 100 nW receptively. These results correspond to a 21 dB improvement in the SNR value as predicted in Fig. 1(f).

### B. Unregulated Voltage Source

The results for the case of unregulated voltage source can be considered as an emulation of a telemetry system that is powered by an energy harvester. At high levels of transmit power the BER tends to zero which is similar to the results shown in 6(c). At a specific level of power (*P*_*T*_ = 4.1 nW) the performance is almost comparable to *P*_*T*_ = 50.1 nW when the power source is regulated, however, the performance gets worse as *P*_*T*_ is reduced to 1 nW.

## V. Conclusion

In this paper, we showed the feasibility of sub-nano-watt in-vivo telemetry links in a water-medium for implantation depths greater than 10 cm. The experimental system comprised of a millimeter-scale piezoelectric crystal, a standard digital pulse-width-modulator, a commercially available echoscope, and a linear array ultrasound transducer. We also presented a simple and yet robust decoding algorithm and we characterized the B-scan telemetry performance using reader sensitivity and bit-error rates. Although the data rates achieved using the proposed scheme are limited by the frame rate of the imager, given modern ultrasound systems [1] that enable imaging at 10 000 fps the data rates using B-scan telemetry scale accordingly without affecting the quality (BER) of the communication link. Future work will include in-vivo validation of the proposed B-scan telemetry using animal models and for applications like orthopedic implant monitoring [9]–[11].

## Supplementary Material

supp1-3053174

## Figures and Tables

**TABLE I:** Comparison of This Work With Previous Related Approaches in Terms of Transmit Power and BER

Ref.	Transducer Size	Pulse Rate	Transmit Power/Voltage	Normalized Power	BER
[12]	2 *mm*	800 Kbps	18 *μM*	22.5 pJ/bit	10^−2^
[17]	2 *mm*	200 Kbps	4 V	-	10^−4^
[19]	1.67 *mm*^3^	125 KHz	600 *μW*	4.8 nJ/bit	10^−4^
[20]	0.045 *mm*^3^	100 Kbps	*177 μW*	1.77 nJ/bit	10^−4^
[21]	9.5 *mm*	28.12 Mbps	25 V	-	0.13
[22]	19 mm	70–700 Kbps	8–40 *μW*	57.14 pJ/bit	10^−4^
[23]	741 *mm*^2^	20 Mbps	-	-	10^−4^
[18]	3.5 *mm*	0.6 Kbps	0.38 *mW*	633.3 pJ/bit	10^−5^
This Work	1 *mm*	27bps	0.83 nW/0.166 V	30.7 pJ/bit	0.22
This Work	1 *mm*	27bps	4.1 nW/1.3 V	151.8 pJ/bit	10^−2^

## Impact Statement—

Reliable, in-vivo ultrasonic telemetry links can be designed using sub-nanowatts of transmission power using a B-scan ultrasound imaging system.
