# Supplementary material for: Sub-Nanowatt Ultrasonic Bio-Telemetry Using B-Scan Imaging
Source: IEEE Open J Eng Med Biol. 2021 Jan 20;2:17–25. doi: 10.1109/OJEMB.2021.3053174 (PMC7978362; doi:10.1109/OJEMB.2021.3053174)
Supplement: Extended experimental results for B-scan telemetry showing sample images for interrogation at different depths and transmission pulse rates. [file supp1-3053174.pdf]

# Supplementary Materials

## Sub-Nano-Watt Ultrasonic Bio-Telemetry using B-scan Imaging

Sri Harsha Kondapalli, *Student Member, IEEE,* and Shantanu Chakrabartty, *Senior Member, IEEE*

### S1. Extended Results

In this work, B-scan telemetry is validated at depths beyond 10 cm and Fig. S1 (a) shows the experimental setup for depths corresponding to 2 cm, 6 cm, 8 cm and 10 cm respectively. Sample B-scan images gathered at each setting is presented in Fig. S1 (b) and the filtered image data is shown in Fig. S1 (c). Note that the amplitude of transmit pulse is set to $V_T = 3.3\ V$ which corresponds to $P_T = 0.3\ \mu W$ . Also sample data for different transmit pulse rate is presented in Fig. S2 (a)-(b) when the interrogation depth is set to 2 cm and 4 cm respectively. The color map for the presented data is shown in the inset of Fig. S2. Fig. S3 shows the raw images form the B-scan telemetry experiments gathered at different instances of time and the consistency in the implant location in the acquired images suggest the repeatability of bench top experiments.

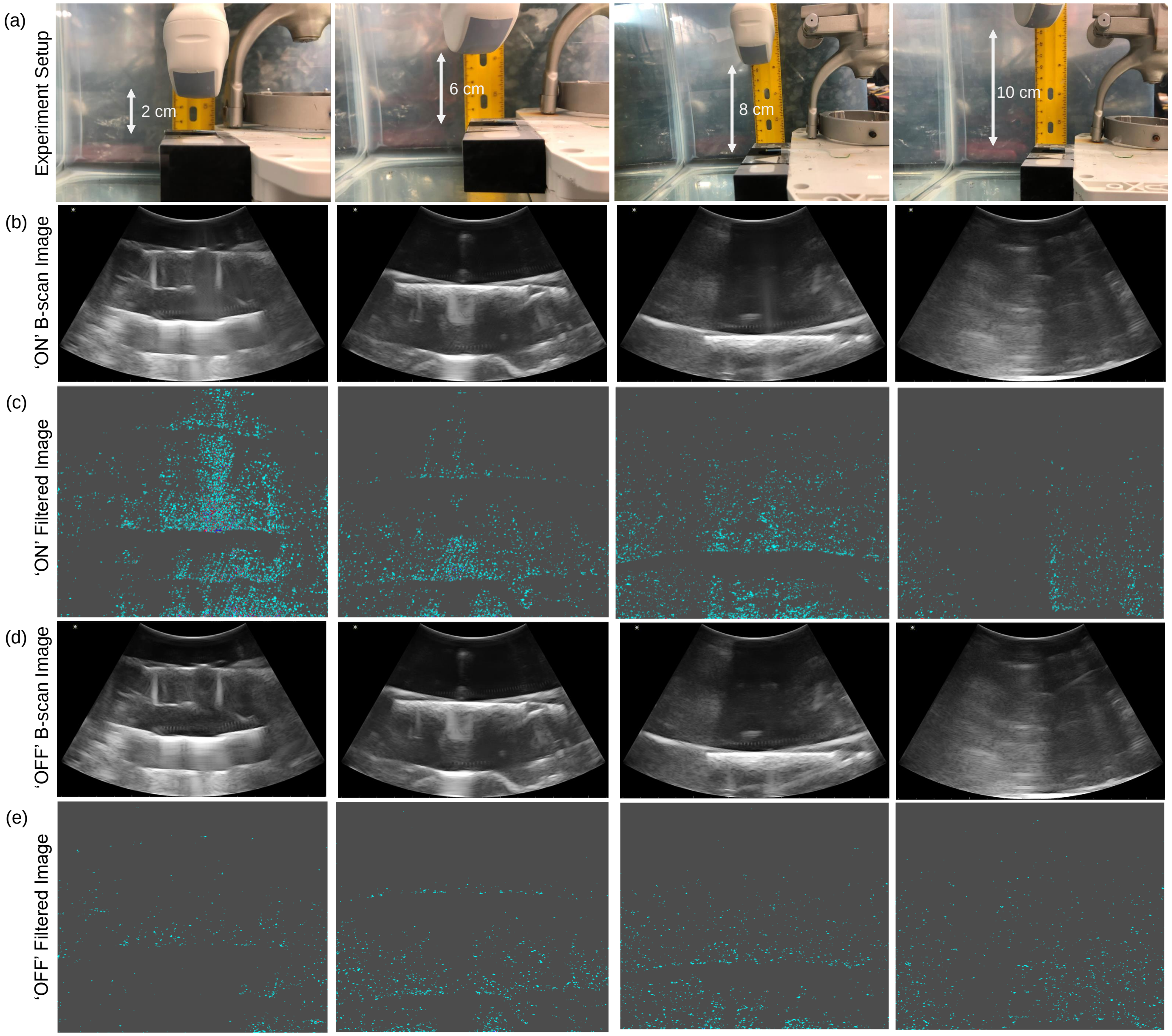


Fig. S1. (a) Experimental setup and (b), (d) sample B-scan telemetry data for ON/OFF states at interrogation distance of 2 cm, 6 cm, 8 cm and 10 cm is shown. Also corresponding filtered B-scan data for ON/OFF state is shown in (c), (e).

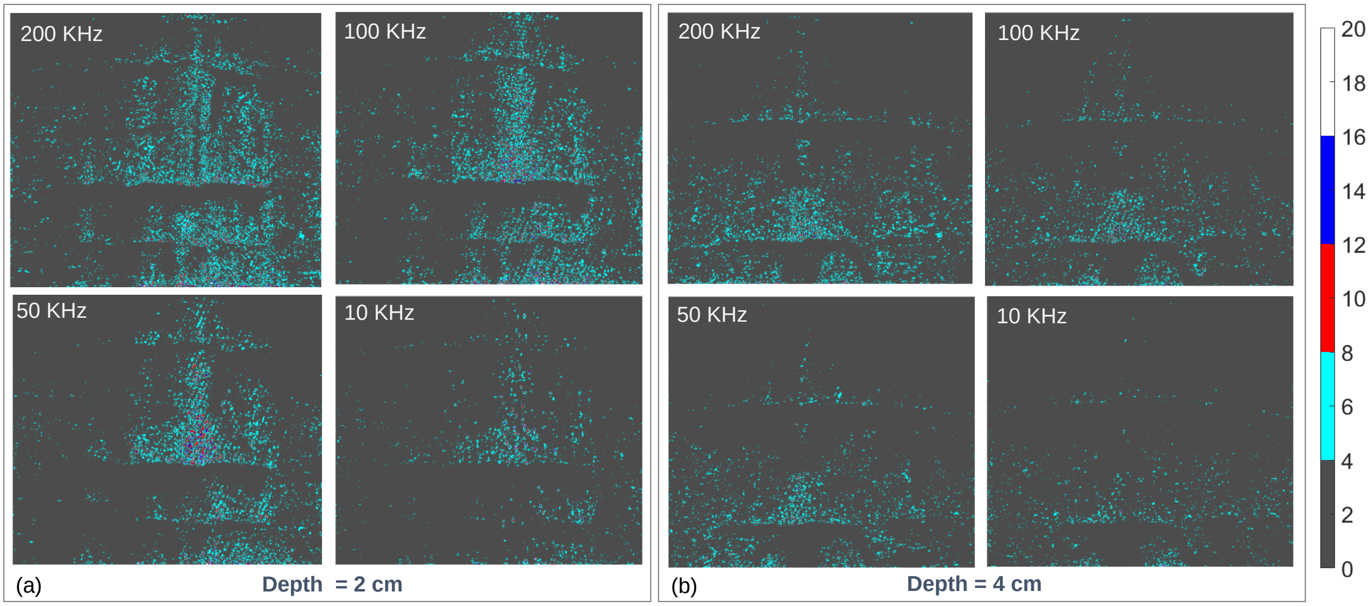


Fig. S2. Sample B-scan telemetry data as the ultrasound pulse rate (Bandwidth) of the transmission pulse is varied from 10 KHz to 200 KHz at interrogation depths of (a) 2 cm and (b) 4 cm respectively.

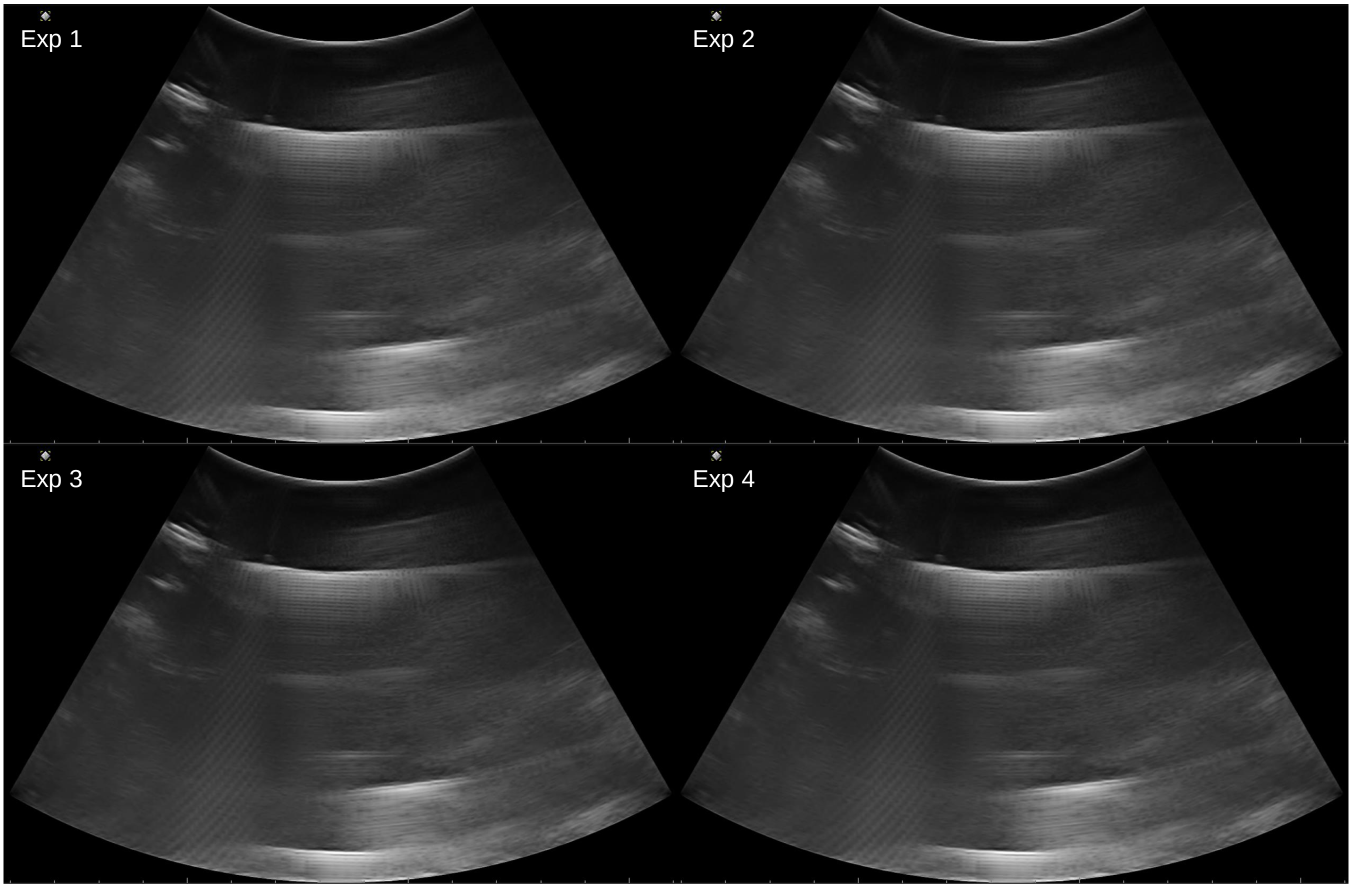


Fig. S3. Sample B-scan telemetry data for fixed transmit power and depth setting at different instances of time shows repeatability of the bench top experiments.
